# Advanced statistics identification of participant and treatment predictors associated with severe adverse effects induced by fluoropyrimidine-based chemotherapy

**DOI:** 10.1007/s00280-023-04538-3

**Published:** 2023-05-10

**Authors:** Samantha K. Korver, Joanne M. Bowen, Rachel J. Gibson, Imogen A. Ball, Kate R. Secombe, Taylor J. Wain, Richard M. Logan, Jonathan Tuke, Kelly R. Mead, Alison M. Richards, Christos S. Karapetis, Dorothy M. Keefe, Janet K. Coller

**Affiliations:** 1 Discipline of Pharmacology, School of Biomedicine, The University of Adelaide, L2 Helen Mayo South, Adelaide, SA 5000 Australia; 2 Discipline of Physiology, School of Biomedicine, The University of Adelaide, Adelaide, Australia; 3 School of Allied Health Science and Practice, The University of Adelaide, Adelaide, Australia; 4 Adelaide Dental School, The University of Adelaide, Adelaide, Australia; 5 School of Mathematical Sciences, The University of Adelaide, Adelaide, Australia; 6 Flinders Medical Centre, Bedford Park, Australia; 7 Flinders University, Bedford Park, Australia; 8 Discipline of Medicine, The University of Adelaide, Adelaide, Australia

**Keywords:** Fluoropyrimidine-based chemotherapy, Adverse effects, Risk, Logistic modeling, Bayesian network analysis

## Abstract

**Purpose:**

Adverse effects following fluoropyrimidine-based chemotherapy regimens are common. However, there are no current accepted diagnostic markers for prediction prior to treatment, and the underlying mechanisms remain unclear. This study aimed to determine genetic and non-genetic predictors of adverse effects.

**Methods:**

Genomic DNA was analyzed for 25 single nucleotide polymorphisms (SNPs). Demographics, comorbidities, cancer and fluoropyrimidine-based chemotherapy regimen types, and adverse effect data were obtained from clinical records for 155 Australian White participants. Associations were determined by bivariate analysis, logistic regression modeling and Bayesian network analysis.

**Results:**

Twelve different adverse effects were observed in the participants, the most common severe adverse effect was diarrhea (12.9%). Bivariate analysis revealed associations between all adverse effects except neutropenia, between genetic and non-genetic predictors, and between 8 genetic and 12 non-genetic predictors with more than 1 adverse effect. Logistic regression modeling of adverse effects revealed a greater/sole role for six genetic predictors in overall gastrointestinal toxicity, nausea and/or vomiting, constipation, and neutropenia, and for nine non-genetic predictors in diarrhea, mucositis, neuropathy, generalized pain, hand–foot syndrome, skin toxicity, cardiotoxicity and fatigue. The Bayesian network analysis revealed less directly associated predictors (one genetic and six non-genetic) with adverse effects and confirmed associations between six adverse effects, eight genetic predictors and nine non-genetic predictors.

**Conclusion:**

This study is the first to link both genetic and non-genetic predictors with adverse effects following fluoropyrimidine-based chemotherapy. Collectively, we report a wealth of information that warrants further investigation to elucidate the clinical significance, especially associations with genetic predictors and adverse effects.

**Supplementary Information:**

The online version contains supplementary material available at 10.1007/s00280-023-04538-3.

## Introduction

Fluoropyrimidine-based chemotherapy regimens are commonly administered for the systemic treatment of solid tumors of the breast, colon and upper gastrointestinal (GI) tract [1]. Often combined with other chemotherapies such as oxaliplatin and irinotecan, fluoropyrimidine-based chemotherapy regimens inhibit DNA and RNA synthesis in highly proliferative tumor cells [2]. The non-selective targeting of highly proliferative cells within the basal epithelial and mucosal membrane of the GI tract as well as hematopoietic stem cells within the bone marrow [3], leads to highly prevalent GI (26–50% [4]) and hematopoietic (70% [5]) adverse effects. These adverse effects include, but are not limited to, nausea, vomiting, mucositis, diarrhea and neutropenia [6]. In addition, adverse effects such as generalized pain, neuropathy, fatigue, hand–foot syndrome, skin toxicity and cardiotoxicity may also occur during treatment with fluoropyrimidine-based chemotherapy regimens [6].

People with cancer receiving fluoropyrimidine-based chemotherapy regimens are not at equal risk of developing adverse effects. A key research emphasis has been on discovering biomarkers to identify those most at risk of developing adverse effects prior to commencing treatment. This is particularly important for the ~ 30% of people who develop severe adverse effects (graded ≥ 3 on the National Cancer Institute’s Common Terminology Criteria for Adverse Events (CTCAE) [7]) in the first six months of treatment [8]. For this subset of people, supportive care measures do not adequately manage symptoms and extreme interventions including treatment delays, dose reductions, hospitalization and early treatment cessation are necessary to effectively manage adverse effects [3]. Current clinically approved biomarkers for severe fluoropyrimidine-based chemotherapy regimens adverse effects include single nucleotide polymorphisms (SNPs) within the *DPYD* gene [9], a gene that encodes for dihydropyrimidine dehydrogenase enzyme required for the metabolism of 5-fluorouracil (5-FU). Similarly, SNPs within key inflammatory genes, which mediate the innate immune response initiated by the non-specific targeting of fluoropyrimidine-based chemotherapy regimens, have also been identified as potential adverse effect risk markers. For example, promoter SNPs in interleukin-1 beta (*IL1B*) and tumor necrosis factor alpha (*TNF*) were related to mucositis after 5-FU treatment in a Japanese population [10]. Whilst our pilot study in an Australian White population that explored a more comprehensive panel of immune and pain-related SNPs revealed risk of severe GI adverse effects after 5-FU was related to *TNF* and toll-like receptor 2 (*TLR2*) SNPs, together with cancer type, a non-genetic predictor [11].

Despite the potential that the FDA-approved *DPYD*, and immune genetic biomarkers, show in becoming clinically translatable biomarkers, both are limited in their ability to identify those at risk of severe adverse effects. For example, *DPYD* variant alleles are present in low frequencies (< 5%) within some ethnicities [9] and, therefore, cannot account for the ~ 30% of severe adverse effects. Similarly, there have been inconsistencies reported to date within the literature pertaining to the possible significance of immune SNPs [10–12]. As such, a new approach of analysis not only combining approved and novel genetic biomarkers for fluoropyrimidine-based chemotherapy severe adverse effects, but also including individual non-genetic demographic, clinical and treatment predictors to increase overall predictive potential is required. These non-genetic parameters include for example age, sex [13] cancer type [11] and 5-FU chemotherapy regimen type [14].

One potential approach that can interrogate the impact of these multiple predictors on the overall risk of adverse effects and the severity of those effects with fluoropyrimidine-based chemotherapy regimens is a layered one. In this way, statistical analysis with increasing stringency, starting with bivariate analysis, followed by logistic modeling and finally Bayesian network analysis is undertaken. Each of these analyses have strengths, and collectively, can indicate associations between predictors and firstly the risk of any adverse effects, and finally those predictors associated with severe adverse effects. Therefore, in light of the inconsistencies of previous studies and the power of combined analytical approach, the aim of this study was to both validate and expand our pilot study findings regarding predictive genetic and non-genetic predictors of fluoropyrimidine-based chemotherapy regimens adverse effects in a group of Australian White participants with breast, colorectal or upper GI solid tumors.

## Materials and methods

### Ethics approval and study design

This retrospective study was approved by both the Southern Adelaide Clinical Human Research Ethics Committee (HREC/12/SAC/519) and the Royal Adelaide Hospital Human Ethics Committee (SSA/14/RAH/519) in Adelaide, Australia. All participants provided informed verbal and written consent prior to participating in accordance with the Declaration of Helsinki.

### Participant recruitment, saliva collection and clinical record data collection

From the pharmacy dispensary records, 243 participants who had received fluoropyrimidine-based chemotherapy regimens for treatment of breast, colorectal or upper GI solid tumors between January 2012 and January 2018 at the Flinders Medical Centre and the Royal Adelaide Hospital, Adelaide, Australia, were invited to participate in the study via a letter. Of the 170 who responded, telephone screening was conducted (JC) to provide verbal consent and remove participants for any of the study exclusion criteria: not fluent in English language, not self-reporting as Australian White, having a pre-existing medical condition associated with GI damage, still receiving chemotherapy treatment, unwilling to provide a saliva sample or have their clinical records reviewed. For the final 155 participants, saliva was collected using an at-home collection kit (DNA Genotek, Kanata, Canada) and returned via mail together with the written participation consent form. Following receipt of the saliva sample and written consent, their clinical records (paper-based) were reviewed by one person (SK) and randomly cross-checked by another independent person (IB) to consistently obtain demographics, comorbidities, type of cancer and fluoropyrimidine-based chemotherapy regimen and adverse effect data. The adverse effects documented were overall GI toxicity, diarrhea, nausea and/or vomiting, mucositis, constipation, neuropathy, generalized pain, hand–foot syndrome, skin toxicity (rash or alopecia), cardiotoxicity (palpitations), fatigue and neutropenia. For each of these, data was recorded as both a categorical (yes/no) occurrence of any grade, and the highest grade/severity (against the National Cancer Institute’s common terminology criteria for adverse events (NCI CTCAE) v 4.03 or 5.0 [7]) experienced over the duration of their chemotherapy.

### Genetic analysis

Genomic DNA was isolated from participant saliva using the prepIT-L2P DNA extraction kit (DNA Genotek). DNA concentration and purity were quantified by spectrophotometry and sent to the Australian Genome Research Facility (AGRF, Brisbane, Australia) for genetic analysis. Genotyping for 21 SNPs in the following genes was conducted using a previously established customized Agena MassArray assay [11]: *IL1B* (rs16944, rs1143627, rs1143634), *IL2* (rs2069762), *IL6* (rs10499563), *IL10* (rs1800871, rs1800896), *IL6R* (rs8192284, now merged to rs2228145), *TGFB1* (rs11466314, rs1800469), *TNF* (rs1800629), *TLR2* (rs3804100), *TLR4* (rs4986790, rs4986791), *MD2* (*LY96*, rs11466004), *MYD88* (rs6853), *BDNF* (rs6265), *CRP* (rs2794521), *CASP1* (rs580253), *CASP5* (previously designated as a *CASP1* variant, rs554344) and *OPRM1* (rs1799971). In addition, genotyping for 4 SNPs in the *DPYD* gene (rs1801158, rs2297595, rs3918290 and rs67376798) was conducted using commercially available TaqMan™ SNP genotyping assays (ThermoFisher Scientific, Thebarton, Australia) with iTaq Universal Probes Supermix (Bio-Rad Laboratories, Gladesville, Australia).

### Statistical analysis

Hardy–Weinberg equilibrium analysis was used to ascertain whether the observed genotype frequencies in participants differed from expected (false discovery rate (FDR)-corrected *P *values < 0.05). Fisher’s exact tests were used to determine if the variant (alternate) allele frequencies of SNPs in the participant cohort was different to the 1000 genomes European population reported on dbSNP (https://www.ncbi.nlm.nih.gov/snp/ accessed on 31st October 2022, FDR-corrected *P* values < 0.05). Power analysis using our pilot model confirmed that 150 participants would provide 99% power at alpha 0.05 to detect the same prediction of GI toxicity.

Due to the complex mechanisms driving the development of adverse effects with fluoropyrimidine-based chemotherapy regimens, three different methods of statistical analysis, each with increasing stringency were utilized. For all analysis, chemotherapy regimens were grouped as 5-FU and leucovorin, capecitabine (alone or with oxaliplatin) or 5-FU+ (5-FU in combination therapies of ECF, EOF, FEC and FOLFOX). All genetic predictors were recoded for analysis with regard to the number of wild-type (WT, reference) alleles and analyzed as nominal categorical predictors. All statistical analysis was conducted using R [15] in RStudio [16] with default standard packages expect for blearn [17] which was necessary for the Bayesian network analysis. Firstly, bivariate analysis using individual logistic regression examined the impact of participants’ genetic (individual SNP rs identifiers), and non-genetic (demographics (age, body surface area, sex), cancer type, fluoropyrimidine-based chemotherapy regimen type, and comorbidities) hereafter referred to as predictors, on the occurrence of adverse effects. Associations between adverse effects (categorical data) and between all other genetic and non-genetic predictors were also examined with Cramér’s V analysis. This is based on a *χ*^2^ statistic and has values between 0 and 1 with 0 indicating no association and values close to 1 indicating a strong association, where *P* < 0.05 was considered significant.

The second method used was logistic regression modeling with step-wise approach of model building to examine predictors of each adverse effect that was partly informed by the bivariate analysis. In brief, each predictor was added to investigate increased strength of the model. Once a predictor was added, a step-back was performed to ascertain if the model could be further improved by removing a previously added predictor. Predictors were added or removed based on Akaike information criterion (AIC). This process built the strongest predictive risk model for each adverse effect. For each predictor in the risk model, we determined if each predictor increased or decreased risk for that adverse effect and also described the relationship in terms of magnitude (the coefficient of probability versus the test case (change in logit, 95% confidence intervals)) and significance (*P* value). Receiver operator characteristic (ROC) curves were obtained by plotting the sensitivity (true positive) of the model against 1-specificity (false positive) of the model [18]. The percentage area under the curve (% AUC) of the ROC curve was then used to assess the ability of the models to predict each adverse effect with the value indicating the percentage of participants who were correctly classified by the model as having each adverse effect. For all adverse effects, three ROC curves were examined: one with genetic predictors alone; one with non-genetic predictors alone; and one with all the predictors.

Finally, to simultaneously investigate associations between adverse effects, the severity/grade of that effect and all collated predictors, Bayesian network analysis was employed [19]. Potential predictive factors from each participant were separated into four tiers based on the type of predictor information as follows:

- *Tier 1*—sex, genotype of each SNP.
- *Tier 2*—comorbidities (other diseases) and lifestyle factors (smoker status, alcohol use).
- *Tier 3*—type of fluoropyrimidine-based chemotherapy regimen and cancer, treatment hospital.
- *Tier 4*—occurrence and severity/grade of each adverse effect.

Bayesian network analysis was run using a score-based method of learning with Hill-Climbing methodology and Bayesian information criterion (BIC) to generate the best direct acyclic graph (DAG), with the lowest BIC score identifying the best DAG. Within the DAG, each predictor was represented as a node and the interconnecting lines or edges revealed the relationship between each predictor. A maximum likelihood estimation (MLE) was then used to determine the strength of the relationship with generated *P* values FDR-corrected to adjust for multiple comparisons.

## Results

### Participant demographics, comorbidities and incidence/severity of adverse effects

Demographic and treatment information for the 155 participants are shown in Table 1. The majority of participants were female (63%) and recruited from the Flinders Medical Centre (69%). Colorectal cancer (*n* = 73) was the most common cancer type and the most common fluoropyrimidine-based chemotherapy regimen was the combination 5-FU+ (ECF, EOF, FEC, FOLFOX) regimens (*n* = 114). Participant comorbidities included alcohol use, arthritis, osteoarthritis, asthma, cardiovascular disease, gastroesophageal reflux disease (GORD), hypertension, hyper- and hypo-thyroidism, osteoporosis, type 2 diabetes mellitus and smoker status (Supplementary Table 1). The most common comorbidities were regular alcohol use during treatment (*n* = 93, 60%) followed by hypertension (*n* = 46, 30%). With regards to the incidence and severity of each adverse effect (based on the NCI CTCAE v 4.03 or 5.0, Supplementary Table 2), all participants that required an intervention (e.g. dose reduction, treatment delay, hospitalization or early treatment cessation) or reported one or multiple grade 3 adverse effects were classified as having severe adverse effects. Diarrhea was the most common severe adverse effect (12.9% grade 3), and also the adverse effect responsible for the majority of dose reductions (6.5%), hospitalizations (4.5%) and early treatment cessations (3%). No grade 4 adverse effects were observed and the study design precluded reporting of grade 5 (death) adverse effects.

**Table 1:** Participant demographics and treatment information

Demographic/treatment information	Total
Sex, *n* (%)	
Male	57 (37)
Female	98 (63)
Treatment hospital, *n* (%)	
Flinders Medical Centre	107 (69)
Royal Adelaide Hospital	48 (31)
Cancer type, *n* (%)	
Breast	72 (46)
Colorectal	73 (48)
Upper gastrointestinal	10 (6)
Fluoropyrimidine-based chemotherapy regimen, *n* (%)	
5-FU+ leucovorin	23 (15)
Capecitabine (alone or with oxaliplatin)	18 (12)
5-FU+ (ECF, EOF, FEC, FOLFOX)^a^	114 (74)
Number of chemotherapy cycles, median (range)	6 (2–30)
Age at treatment, median (range)	64 (28–86)
Surgery, *n* (%)	
None	9 (6)
Pre-treatment	129 (83)
Post-treatment	12 (8)
Pre- + post-treatment	5 (3)
Radiotherapy, *n* (%)	
None	74 (48)
Pre-treatment	16 (10)
Post-treatment	65 (42)

^a^ECF: epirubicin, cisplatin and 5-FU; EOF: epirubicin, oxaliplatin and 5-FU; FEC: 5-FU, epirubicin and cyclophosphamide; FOLFOX: 5-FU, leucovorin and oxaliplatin

### Occurrence of genetic variability

Genomic data for each SNP was available for between 150 and 154 participants (Supplementary Table 3). Hardy–Weinberg equilibrium analysis was not significant for any SNP (*P* > 0.51, FDR-corrected *P* > 0.92). Two SNPs in the *TGFB1*, rs11466314 and *DPYD*, rs3918290 genes were non-polymorphic in the cohort and not evaluated in further analysis. The variant (alternate) allele frequencies of all SNPs were similar to the European population reported on dbSNP (*P* > 0.069, FDR-corrected *P* > 0.92, Supplementary Table 3).

### Associations of predictors with adverse effects

#### Bivariate analysis

Table 2 summarizes the impact of participants genetic and non-genetic predictors, on the occurrence of the adverse effects examined with individual logistic regression analyses. Eight genetic and 12 non-genetic predictors were associated with at least one adverse effect, with different fluoropyrimidine-based chemotherapy regimens being the predictor associated with the most adverse effects (diarrhea, mucositis, constipation, neuropathy, generalized pain, hand–foot syndrome and fatigue).

**Table 2:** *P* values of significant associations obtained from individual logistic regression analyses between adverse effects and participant genetic (gene name, single nucleotide polymorphism (SNP) rs identifier), and non-genetic (demographics, cancer and fluoropyrimidine-based chemotherapy regimen types, and comorbidity) predictors

	Diarrhea	Nausea and/or vomiting	Mucositis	Constipation	Neuropathy	Pain^a^	Hand–foot syndrome	Skin toxicity	Cardiotoxicity	Fatigue	Neutropenia
*IL6R*, rs8192284				0.049							
*TGFB1*, rs1800469		0.022									0.007
*TLR4*, rs4986790		0.040					0.020				
*TLR4*, rs4986791					0.046		0.021				
*MD2* (*LY96*), rs11466004			0.011							0.003	
*BDNF*, rs6265											0.037
*DPYD*, rs67376798										0.010	
*DPYD*, rs2297595				0.038							
Sex	0.001				< 0.0001		0.017				
Treatment hospital	0.027		< 0.0001		0.002				0.0001	< 0.0001	
Cancer type	< 0.0001				< 0.0001		0.010				
Fluoropyrimidine-based chemotherapy regimen	0.023^b^		0.037^c^	0.019^c^	0.002^d^	0.013^c^	0.009^b^			0.040^c^	
Age at treatment				0.024					0.012		
Body surface area	0.005				0.021						
Alcohol use				0.025		0.047					
Arthritis					0.019					0.009	
Osteoarthritis						0.034					
Hypothyroidism						0.018					
Osteoporosis					0.019						
Type 2 diabetes mellitus									0.002		
Smoker status				0.021				0.008		0.0006	

^a^Generalized pain

^b^5-FU and leucovorin, and capecitabine (alone or with oxaliplatin)

^c^5-FU+ regimens

^d^5-FU+ regimens and capecitabine (alone or with oxaliplatin)

**Fig. 1:**
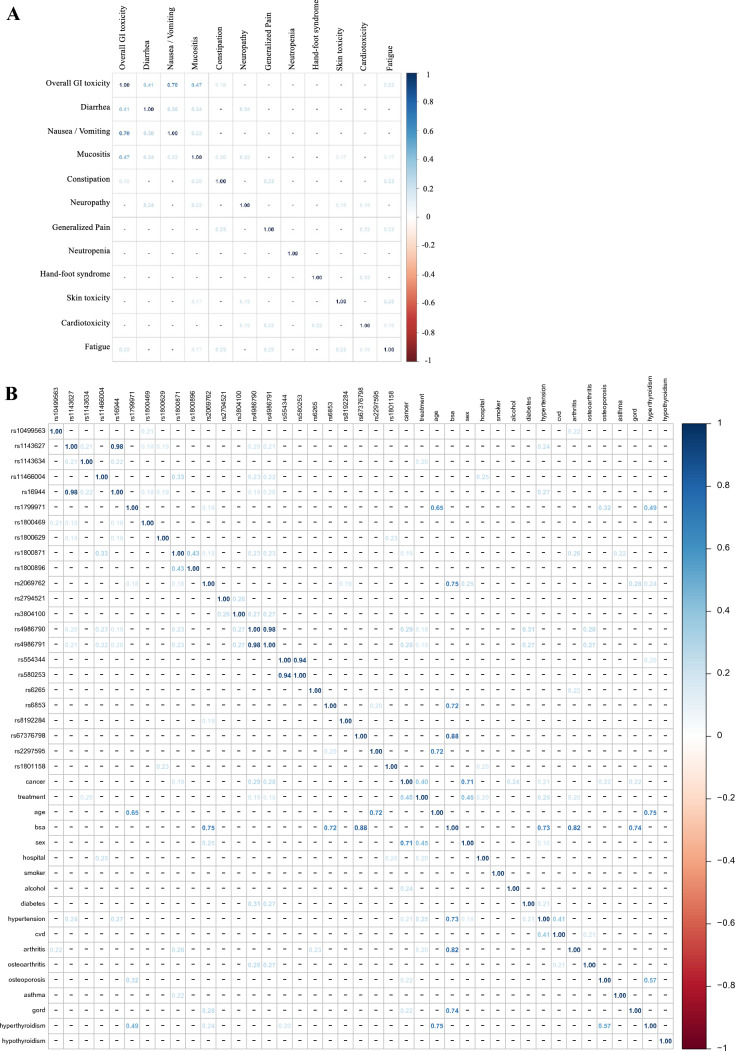
Heatmap of significant associations between **A** adverse effects and **B** genetic and non-genetic predictors, with blue colours indicating positive associations and Cramér’s V numbers indicating the strength of the associations. Predictor abbreviations are: cancer: cancer type; treatment: fluoropyrimidine-based chemotherapy regimen type; bsa: body surface area; hospital: treatment hospital; cvd: cardiovascular disease; gord: gastroesophageal reflux disease

Associations between the different adverse effects were also determined and presented as a heatmap with Cramér’s V numbers indicating the strength of the association (Fig. 1a). All adverse effects except neutropenia were associated with at least one other adverse effect. These strongest associations were observed between the overall GI toxicity and the different types of GI toxicity (Cramér’s V 0.47–0.7), with all other associations being much weaker (all less than 0.24). Similar analysis between all other genetic and non-genetic predictors also revealed associations of ranging strength displayed as another heatmap with Cramér’s V numbers (Fig. 1b). Amongst these, strong genetic predictor associations were observed between *IL1B* rs16944 and rs1143627 (0.98), *TLR4* rs4986790 and rs4986791 (0.98), and *CASP1* rs554344 and *CASP5* rs580253 (0.94). Strong genetic-non-genetic predictor associations were also detected, for example several SNPs and body surface area: *DPYD* rs67376798 (0.88), *IL2* rs2069762 (0.75), and *BNDF* rs6853 (0.72). Finally strong non-genetic predictor associations were also observed, for example several comorbidities and body surface area: arthritis (0.82), GORD (0.74), and hypertension (0.73).

#### Logistic regression modeling

Details of the predictors included in the final models for each adverse effect are listed below as either associated with a decreased or an increased probability of the participant having experienced the adverse effect. Individual change in logit, 95% confidence intervals and *P* values for each predictor are provided in Supplementary Table 4.

- Overall GI toxicity:
  - Decreased probability: treatment hospital, Royal Adelaide Hospital.
  - Increased probability: *TGFB1* rs1800469 1 copy wild-type (WT, reference) allele; *TGFB1*, rs1800469 2 copies WT allele.

- Diarrhea:
  - Decreased probability: treatment hospital, Royal Adelaide Hospital.
  - Increased probability: *IL10*, rs1800871 2 copies WT allele; colorectal cancer; gastric cancer; *IL10,* rs1800871 1 copy WT allele.

- Nausea and/or vomiting:
  - Decreased probability: *TLR4*, rs4986790 1 copy WT allele; *TLR4*, rs4986790 2 copies WT allele; increasing body surface area.
  - Increased probability: *TGFB1*, rs1800469 2 copies WT allele; *IL6*, rs10499563 1 copy WT allele; *TGFB1*, rs1800469 1 copy WT allele; *IL6*, rs10499563 2 copies WT allele.

- Mucositis:
  - Decreased probability: treatment hospital, Royal Adelaide Hospital.
  - Increased probability: *MD2* (*LY96*), rs11466004 2 copies WT allele.

- Constipation:
  - Decreased probability: *BDNF*, rs6265 1 copy WT allele; *BDNF*, rs6265 2 copies WT allele; capecitabine chemotherapy (alone or with oxaliplatin).
  - Increased probability: *DPYD*, rs2297595 1 copy WT allele; *DPYD*, rs2297595 2 copies WT allele; *IL6R*, rs81922841 1 copy WT allele; alcohol use; *IL6R* (rs81922841) 2 copies WT allele; 5-FU+ chemotherapy (ECF, EOF, FEC, FOLFOX); smoker; non-smoker.

- Neuropathy:
  - Decreased probability: treatment hospital, Royal Adelaide Hospital.
  - Increased probability: 5-FU+ chemotherapy (ECF, EOF, FEC, FOLFOX); colorectal cancer; capecitabine chemotherapy (alone or with oxaliplatin); gastric cancer.

- Generalized pain:
  - Increased probability: 5-FU+ chemotherapy (ECF, EOF, FEC, FOLFOX); capecitabine chemotherapy (alone or with oxaliplatin); hypothyroidism; alcohol use.

- Neutropenia:
  - Decreased probability: *TLR4*, rs4986790 2 copies WT allele; *TLR4*, rs4986790 1 copy WT allele; *CASP5*, rs554344 1 copy WT allele; *CASP5*, rs554344 2 copies WT allele; *TGFB1*, rs1800469 1 copy WT allele.
  - Increased probability: *BDNF*, rs6265 2 copies WT allele; *BDNF*, rs6265 1 copy WT allele; *TGFB1*, rs1800469 2 copies WT allele.

- Hand–foot syndrome:
  - Decreased probability: *TLR4*, rs4986790 1 copy WT allele; *TLR4*, rs4986790 2 copies WT allele; 5-FU+ chemotherapy (ECF, EOF, FEC, FOLFOX).
  - Increased probability: *IL10*, rs1800871 2 copies WT allele; capecitabine chemotherapy (alone or with oxaliplatin); *IL10*, rs1800871 1 copy WT allele.

- Skin toxicity:
  - Decreased probability: non-smoker; smoker.

- Cardiotoxicity:
  - Decreased probability: treatment hospital, Royal Adelaide Hospital; *CASP5*, rs554344 2 copies WT allele; CASP5, rs554344 1 copy WT allele.
  - Increased probability: type 2 diabetes mellitus; increasing age at treatment.

- Fatigue:
  - Decreased probability: arthritis; treatment hospital, Royal Adelaide Hospital; non-smoker.
  - Increased probability: *DPYD*, rs67376798 2 copies WT allele; *MD2*, rs11466004 2 copies WT allele; smoker.

**Fig. 2:**
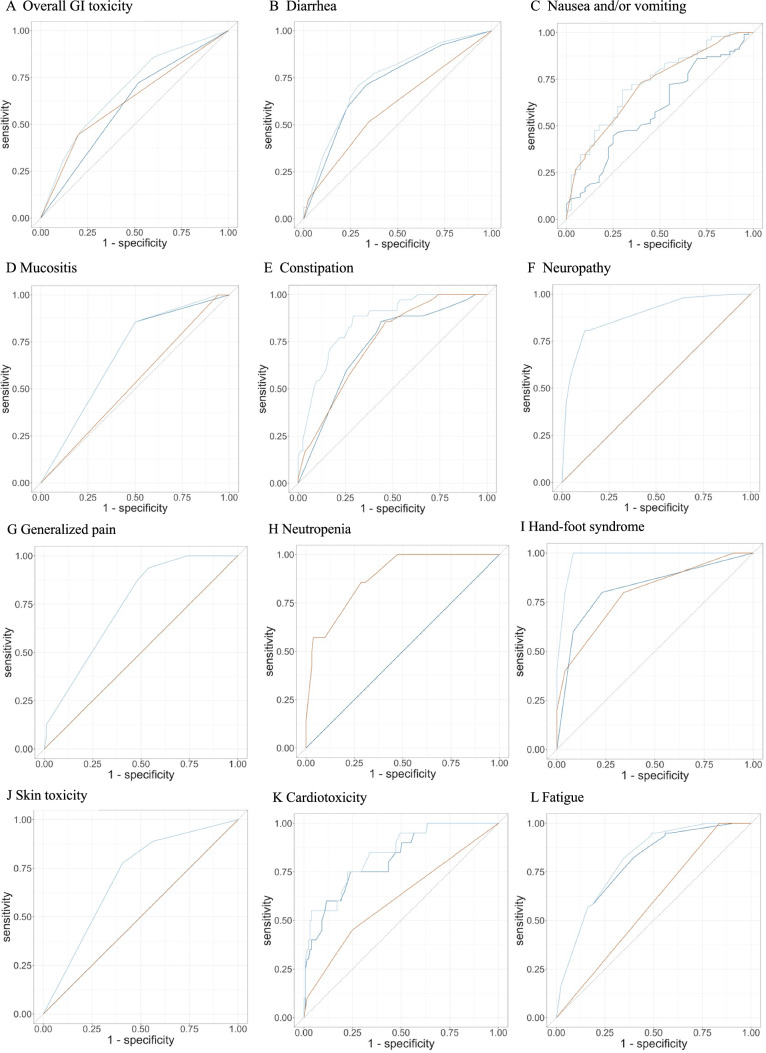
Receiver operator characteristic (ROC) curves of individual logistic regression models identifying associations between each adverse effect and genetic predictors (red line), non-genetic predictors (dark blue line) and all predictors (light blue line). Adverse effects are **A** overall GI toxicity, **B** diarrhea, **C** nausea and/or vomiting, **D** mucositis, **E** constipation, **F** neuropathy, **G** generalized pain, **H** neutropenia, **I** hand–foot syndrome, **J** skin toxicity, **K** cardiotoxicity and **L** fatigue

ROC curves of the individual models incorporating genetic predictors, non-genetic predictors or all predictors for each adverse effect are displayed in Fig. 2A–K. The percentage area under the ROC curves (% AUC) are in Table 3. The highest sensitivity and selectivity of the models was observed for hand-foot syndrome (all predictors, 98% AUC) followed by neuropathy (all predictors, 89% AUC) and neutropenia (all predictors and genetic predictors, 88% AUC).

**Table 3:** Percentage area under the curve (% AUC) values for receiver operator characteristic (ROC) curves of logistic regression models for each adverse effect

Adverse effect type	Genetic predictors	Non-genetic predictors	All predictors
Overall GI toxicity	62	60	68
Diarrhea	60	72	74
Nausea and/or vomiting	71	59	73
Mucositis	53	68	68
Constipation	74	72	85
Neuropathy	–	89	89
Generalized pain	–	74	74
Neutropenia	88	–	88
Hand–foot syndrome	79	82	98
Skin toxicity	–	70	70
Cardiotoxicity	61	81	84
Fatigue	58	79	81

Bayesian network analysis.

**Fig. 3:**
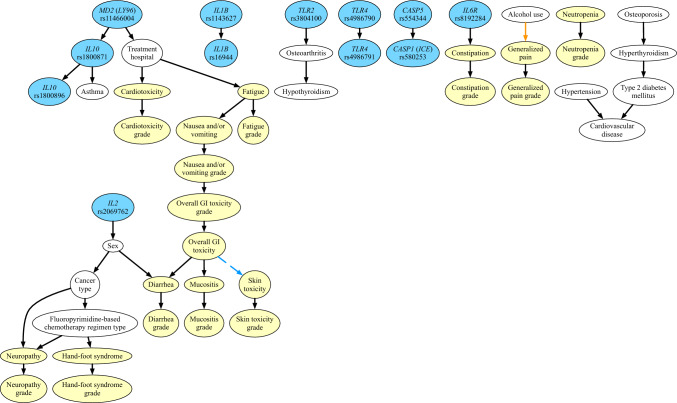
DAG generated by the Bayesian network analysis with significant relationships between predictors from the different tiers of varying strength indicated by the colour of the arrows; black arrows represent *P* values < 0.01, the orange arrow represents a *P* value between 0.01 and 0.05, and the blue dashed arrow represents a *P* value between 0.05 and 0.1. All genetic predictors in the network are shaded in blue, all non-genetic predictors in the network are shaded in white, and the adverse effects and grade/severity of those effects in the network are shaded in yellow

The DAG (Fig. 3) generated by the Bayesian network analysis revealed significant relationships between predictors from the different tiers with varying strength of relationships (*P* values ranged from < 0.01 to 0.1). In brief, eight SNPs were identified in direct relationships: *IL1B*, rs1143627 and *IL1B*, rs16944; *IL2*, rs2069762 and sex (greater frequency of homozygous variant genotype in males); *IL10*, rs1800871 and *IL10*, rs1800896; *IL10*, rs1800871 and asthma; *IL6R*, rs8192284 and constipation; *TLR2*, rs3804100 and osteoarthritis; *TLR4*, rs4986790 and *TLR4*, rs4986791; *MD2* (*LY96*), rs11466004 and *IL10*, rs1800871; and *CASP5*, rs554344 and *CASP1* (*ICE*), rs580253. Other direct relationships were identified between the different adverse effects: fatigue and nausea and/or vomiting; overall GI toxicity and mucositis; overall GI toxicity and diarrhea; and overall GI toxicity and skin toxicity. Direct relationships were also identified between different non-genetic predictors and/or different adverse effects: sex and cancer type (higher percentage males with colorectal and gastric cancer); sex and diarrhea (higher frequency in males); cancer type and fluoropyrimidine-based chemotherapy regimen; cancer type and neuropathy; fluoropyrimidine-based chemotherapy regimen and neuropathy; fluoropyrimidine-based chemotherapy regimen and hand–foot syndrome; treatment hospital and cardiotoxicity; alcohol use and generalized pain; osteoarthritis and hypothyroidism; osteoporosis and hyperthyroidism; hyperthyroidism and type 2 diabetes mellitus; type 2 diabetes mellitus and cardiovascular disease; and hypertension and cardiovascular disease. Finally, direct relationships were observed between each adverse effect and the observed severity/grade of that adverse effect.

## Discussion

This retrospective study investigated the impact of different types of predictors on the occurrence of adverse effects in 155 participants following treatment with fluoropyrimidine-based chemotherapy regimens using three levels of statistical analyses of increasing complexity. The common findings across all analyses were associations between different adverse effects and associations between genetic and non-genetic predictors and adverse effects.

### Bivariate associations between adverse effects and between predictors of adverse effects

We first examined associations between adverse effects following treatment with fluoropyrimidine-based chemotherapy regimens with bivariate analysis given the body of evidence reporting concurrent adverse effects, or symptom clustering, during chemotherapy treatment. The strongest associations were identified between the overall GI toxicity adverse effect and the individual adverse effects (i.e. nausea and/or vomiting, mucositis and diarrhea) that combined to give the overall effect. However, this study also identified other weaker associations. For example, between neuropathy, skin toxicity and cardiotoxicity and other adverse effects. Importantly, the associations between neuropathy and GI effects seen in this study had previously been indirectly linked in a Bayesian network analysis in colorectal cancer patients receiving different chemotherapy regimens [20]. These associations may be explained by common underlying mechanisms for these different adverse effects, such as the increased pro-inflammatory signaling that occurs with GI adverse effects [2] and with neuropathy following chemotherapy [21]. Similarly, the associations between skin toxicity, measured as rash or alopecia in this study, and fatigue, neuropathy and mucositis were expected, given that rash was previously indirectly linked to fatigue, neuropathy and mucositis in a previous Bayesian network analysis [20]. Finally, the links we observed between cardiotoxicity, measured as palpitations in the current study, and generalized pain and fatigue, are in accordance with previous reports linking palpitations with pain [20] and fatigue [20, 22].

Similar to associations between adverse effects, it was important to consider bivariate associations between the investigated genetic and non-genetic predictors prior to logistic regression and Bayesian network analysis to maintain the study power by keeping the number of predictors within the dataset to a minimum. Whilst there were some strong associations detected, for example between the *IL1B* rs16944 and rs1143627 SNPs, between the *DPYD* rs67376798 SNP and body surface area, and between body surface area and arthritis, none of these associations were evident in 100% of the participants. Consequently, we did not remove any predictors from further analysis on the basis of these bivariate associations.

### Logistic regression models associating genetic and non-genetic predictors with adverse effects

Our next step was to use logistic regression to determine which genetic and non-genetic predictors were associated with each adverse effect with % AUC of ROC curves indicating the sensitivity and specificity of the models that included genetic predictors alone, non-genetic predictors alone, and all predictors.

Briefly, the genetic predictors appeared to play a greater role compared to the non-genetic predictors in the adverse effects of overall GI toxicity, nausea and/or vomiting and constipation, as evidenced by the higher % AUC for the ROC curves and were the sole predictors of neutropenia. Consequently, we have limited our discussion to genetic predictors observed in these models.

With regards to GI-related adverse effects and genetic predictors, a *TGFB1* (rs1800469) SNP was common to overall GI toxicity and nausea and/or vomiting models. The variant allele of this SNP has previously been associated with increased anti-inflammatory TGF-β expression compared to the WT allele [23, 24]. Consequently, participants with the WT allele who would be expected to express less TGF-β to explain their increased probability of experiencing these adverse effects. The other SNP associated with increased probability of nausea and/or vomiting was *IL6* rs10499563 which aligns with previous research reporting carriers of the WT allele have increased IL-6 expression [25]. In contrast, it was observed that participants with WT alleles of the *TLR4* SNP rs49986790 had a decreased probability of nausea and/or vomiting. Although there is mixed evidence linking this SNP to changes in function (for review see Ref. [26]), more recently this SNP has been linked to reduced IL-6 plasma expression following mixed chemotherapy regimens including irinotecan and 5-FU [27]. Consequently, WT alleles carriers of *TLR4* may express less IL-6 and have reduced inflammation to reduce the probability of nausea and/or vomiting. Therefore, associations with these genetic predictors support the pro-inflammatory hypothesis underlying occurrence of these GI-related adverse effects [2, 28, 29].

In contrast, it is less clear why SNPs in *IL6R* rs8192284 and *DPYD* rs2297595 were predictors of increased probability, and *BDNF* rs6265 of decreased probability, of constipation. WT alleles of the *IL6R* have been previously reported to cause less soluble plasma IL-6R in comparison to variant alleles [25]. This has the potential to reduce IL-6 signaling and hence, participants with WT alleles would be expected to have decreased probability of experiencing constipation, not an increased probability as observed. In addition, although there have been no reports associating the *DPYD* SNP and constipation, it has been recently linked to other adverse effects, including abdominal pain, oral mucositis and diarrhea [30]. Finally, the association between participants with WT alleles of the *BDNF* rs6265 SNP and higher BDNF secretion [31] having decreased probability of constipation aligns with the role that BDNF plays in GI motility. For example, it has been reported that people with slow-transit constipation, classified by the Rome II criteria and having intestinal transit time of ≥ 96 h, had 62% lower colonic BDNF expression compared to controls [32]. Therefore, due to unclear nature of the impact of some of these genetic predictors, further detailed research is required to further elucidate associations with constipation. This research would also need to investigate the impact of drug-related constipation (e.g. from the use of opioids or anti-motility agents) as an additional predictor for this adverse effect which was not captured in the current study.

Similarly, the underlying mechanisms associating the observed genetic predictors to neutropenia are also unclear in light of previous literature and paucity of reports regarding the functional impact of these SNPs. For example, an increased probability of neutropenia with WT alleles of *BDNF* rs6265 and hence increased BDNF secretion was not expected as BDNF has previously been shown to promote immune recovery after radiation treatment in a pre-clinical model [33]. In contrast, a recent pre-clinical report of *Lacticaseibacillus casei* treatment reducing both 5-FU-induced leukopenia and *TLR4* gene expression in the jejunum [34] may explain why carriers of WT alleles of *TLR4* rs4986790 had a decreased probability of neutropenia. Whilst the link between the *CASP5* rs554344 SNP WT allele and decreased probability of neutropenia may be explained by the key role of caspase-5 in the inflammasome (for review see Ref. [35]). Finally, the observed mixed (decreased and increased) probability of neutropenia in carriers with 1 or 2 copies of WT alleles of *TGFB1* limits meaningful discussion of a possible association.

In contrast to the adverse effects above, non-genetic predictors played a greater role in diarrhea, mucositis, hand–foot syndrome, cardiotoxicity and fatigue models, and were the sole predictors in the neuropathy, generalized pain and skin toxicity models. As most predictors were associated with only one type of adverse effect, we have limited our discussion here to the most common predictors across the adverse effects.

Firstly, the treatment hospital (Royal Adelaide Hospital) was associated with a decreased probability of diarrhea, mucositis, cardiotoxicity, fatigue and neuropathy adverse effects. However, given the retrospective nature of the current study and differences in potential for data collection from clinical records compared to research purposes, we believe this could be explained by underreporting of the adverse effect in records at that hospital, rather than a true difference. Indeed, there is evidence regarding reporting differences between not only health professionals [36], but also between health professionals and self-reporting by people receiving chemotherapy [37] which highlights some of the difficulties in replicating adverse effect data collection and collation across time and clinical settings. Unfortunately, the data collection method of the current study prevents further elucidation, and consequently prospective investigations are required to confirm or reject this possibility.

Of the other non-genetic predictors investigated, type of chemotherapy regimen was also commonly associated with these adverse effects, for example increased probability of neuropathy and generalized pain was observed with 5-FU+ chemotherapy (ECF, EOF, FEC, FOLFOX) and capecitabine (alone or with oxaliplatin) chemotherapy. It is well established that nervous system toxicity is experienced more commonly following certain regimens that contain oxaliplatin and cisplatin platinum-based chemotherapies (for review see Ref. [38]) such as ECF, EOF and FOLFOX. Therefore, an increased probability of neuropathy and generalized pain would be expected to be associated with these regimens.

Finally, smoking status was a common predictor associated with fatigue and skin toxicity adverse effects. With regard to fatigue, our observations that smokers had an increased probability of this adverse effect aligns with a report that long-term fatigue post-chemotherapy was related to smoking [39]. However, the association with skin toxicity remains unclear as both non-smokers and smokers had a decreased probability of this adverse effect compared to ex-smokers.

The overall contribution of all predictors to the adverse effects was also assessed and improved the % AUC of the ROC curves by greater than 10% for the constipation and hand–foot syndrome adverse effects. The possible mechanism underlying the influence of the genetic predictors and constipation was discussed above. In contrast, the association of the non-genetic predictors requires further investigation. For example, the impact of chemotherapy regimen-type predictors with constipation remains unclear but could be explained by different drug classes (e.g. platinum-based, oxaliplatin [40]) in the different regimens. Similarly, the associations of the genetic predictors with hand–foot syndrome, namely the *TLR4* (rs4986790) SNP with decreased probability, and the *IL10* (rs1800871) SNP with increased probability remains unclear with no previous reports in the literature. Whilst the mixed (increased and decreased) probability of association of chemotherapy regimen types with this adverse effect mostly likely is explained by the differing potential of chemotherapeutics to cause this adverse effect (for review see Ref. [41]).

With regards to validating our pilot study findings that identified *TLR2* and *TNF* genetic and colorectal and gastric cancer type non-genetic predictors of overall GI toxicity [11], a combined model in the current study only improved the % AUC of ROC curves from the genetic predictors model by 6% and observed different genetic (*TGFB1*) and non-genetic predictors. Although both were retrospective studies, there were a number of differences in the pilot vs current study that could explain the differences. These included the number of participants (34 vs 155), frequencies of the different cancer types (breast: 38 vs 46%; colorectal: 53 vs 48%; upper gastric 9 vs 6%) and chemotherapy regimen types (5-FU/LV: 17 vs 15%; capecitabine: 15 vs 11%; 5-FU+ : 68 vs 74%), the time when the study was conducted (2009–2012 vs 2012–2018) and recruitment from different treatment hospitals (Flinders Medical Centre vs Flinders Medical Centre and Royal Adelaide Hospital). We believe the latter two factors in particular may explain the differences in models, especially the degree to which the adverse effects were reported and the quality of that reporting in the clinical records as discussed previously.

### Bayesian network associations between predictors and adverse effects

Given the above complexity of understanding both the genetic and non-genetic predictors associated with the adverse effects from the other analysis approaches, it was important to attempt to combine all available information in the current study using the more stringent Bayesian network analysis that also accounted for the severity/grade of the adverse effect. This novel analysis reduced the number of links directly and indirectly with both genetic and non-genetic predictors and included direct links between the different adverse effects and the severity/grade of that effect.

Despite not replicating our pilot findings, the links in the network between some SNPs were expected from previous studies. For example, between *IL1B* rs1143627 and rs16944, *IL10* rs1800871 and rs1800896, *TLR4* rs4986790 and rs4986791, and *CASP5* (previously *CASP1*) rs554344 and *CASP1* rs580253 [42, 43], and were observed, but not specifically reported, in our pilot study cohort [11]. In contrast, the link between the *MD2* (*LY96*) rs11466004 and *IL10* rs1800871 SNPs has not been previously reported. The direct links between adverse effects and predictors, some of which were novel, were minimal and included: treatment hospital and cardiotoxicity/fatigue; *IL6R* rs8192284 and constipation; sex and diarrhea; cancer type and neuropathy; fluoropyrimidine-based chemotherapy regimen type and neuropathy/hand–foot syndrome; alcohol use and generalized pain; and various links between the different adverse effects. Also of note were nodes directly linking different comorbidities which had some basis given our understanding of the predictors of these comorbidities, for example hypertension and cardiovascular disease. Although of great interest, the novelty and descriptive nature of this network (the first Bayesian network to include genetic and non-genetic predictors) limited our ability to compare our network with others in the literature. Consequently, these observations will require future studies to replicate the links observed that would also examine the network stability with bootstrapping or other methods. In addition, as the severity/grade of the adverse effects was not directly linked to other genetic and non-genetic predictors, rather only indirectly linked through the adverse effect itself, the clinical significance of these indirect links with regards to people at risk of grade 3 severe adverse effects requires further elucidation.

### Limitations

The current study observed novel genetic and non-genetic predictors of numerous adverse effects using a layered statistical approach, however, several limitations must be acknowledged. Firstly, as discussed above, this was a retrospective study that mined adverse effect data from clinical records. As such we were limited by the extent and detail in the reporting and as a consequence, cannot be certain that predictors of these adverse effects have been missed in our dataset. However, this is the real world experience. Another limitation due to the participant numbers was that predictors for each adverse effect could not be explored independently for each cancer type, however, this was controlled by inclusion of cancer type as a predictor in the analysis. Further, with regard to the genetic predictors due to the unknown functional impact of some of the SNPs, we chose to analyse these predictors with regard to the number of WT alleles carried by each participant which may have influenced the associations. Together, the overall impact of these limitations cannot be estimated but rather they highlight the challenges faced when performing this type of clinical research. Indeed, there has been extensive discussion in the field for how studies should proceed to allow a more thorough interrogation of predictors of adverse effects [20, 44–46].

### Conclusions

For the first time, the current study has provided a wealth of thought-provoking information on genetic and non-genetic predictors of numerous adverse effects following fluoropyrimidine-based chemotherapy treatment, utilizing various statistical approaches. In particular, the Bayesian network analysis is the first to contain both genetic and non-genetic predictors. Genetic predictors of interest included *TGFB*, *IL6*, *IL6R*, *CASP5*, *BDNF*, *TLR4* and *DPYD* SNPs whilst chemotherapy regimen type was a common non-genetic predictor. Future research is required to replicate and explore the clinical significance of these findings.

## Supplementary Information

Below is the link to the electronic supplementary material.

Supplementary file1 (DOCX 39 KB)

## Data Availability

The datasets generated during and/or analysed during the current study are available from the corresponding author on reasonable request.
