# Supplementary material for: Advanced statistics identification of participant and treatment predictors associated with severe adverse effects induced by fluoropyrimidine-based chemotherapy
Source: Cancer Chemother Pharmacol. 2023 May 10;91(6):507–21. doi: 10.1007/s00280-023-04538-3 (PMC10191967; doi:10.1007/s00280-023-04538-3)
Supplement: Supplementary file 1 — Supplementary file1 (DOCX 39 KB) [file 280_2023_4538_MOESM1_ESM.docx]

Supplementary Information for:

**Advanced statistics identification of participant and treatment predictors associated with severe adverse effects following fluoropyrimidine-based chemotherapy**

Cancer Chemotherapy and Pharmacology

Samantha Korver, Joanne M Bowen, Rachel J Gibson, Imogen Ball, Kate Secombe, Taylor J Wain, Richard M Logan, Jonathan Tuke, Kelly Mead, Alison Richards, Christos S Karapetis, Dorothy Keefe and Janet K Coller

Corresponding Author:

Dr Janet Coller

School of Biomedicine, L2 Helen Mayo South,

University of Adelaide, Adelaide

South Australia, 5000, Australia

Email: [janet.coller@adelaide.edu.au](mailto:janet.coller@adelaide.edu.au)

Supplementary Table 1: Participant comorbidity data from clinical medical records reported as a ‘Yes’, ‘No’ or ‘Not reported’ to reflect occurrence at the time of treatment whilst smoker status was additionally classified as ‘Ex-smoker’ to reflect smoking prior to treatment. Data are n (%).

| Comorbidity | Yes | | No | | Not reported |
| --- | --- | --- | --- | --- | --- |
| Alcohol Use | 93 (60) | | 29 (19) | | 33 (21%) |
| Arthritis | 6 (4) | | 149 (96) | |  |
| Osteoarthritis | 18 (12) | | 137 (88) | |  |
| Asthma | 18 (12) | | 137 (88) | |  |
| Cardiovascular disease | 27 (17) | | 128 (83) | |  |
| Gastroesophageal reflux disease | 28 (18) | | 127 (82) | |  |
| Hypertension | 46 (30) | | 109 (70) | |  |
| Hyperthyroidism | 2 (1) | | 153 (99) | |  |
| Hypothyroidism | 5 (3) | | 150 (97) | |  |
| Osteoporosis | 6 (4) | | 149 (96) | |  |
| Type 2 diabetes mellitus^a^ | 13 (8) | | 142 (92) | |  |
|  | Yes | No | | Ex-smoker | Not reported |
| Smoker status | 19 (12) | 49 (32) | | 58 (37) | 29 (19) |

^a^ Type 2 diabetes mellitus controlled by either diet or medication.

Supplementary Table 2: Incidence and severity of adverse events following fluoropyrimidine-based chemotherapies and subsequent interventions of dose reductions, treatment delays, hospitalization and early treatment cessation as reported in participant clinical records.

|  | Incidence of adverse event, n (%)^a^ | | | | Intervention, n (%) | | | |
| --- | --- | --- | --- | --- | --- | --- | --- | --- |
| Adverse event type | Grade 0 | Grade 1 | Grade 2 | Grade 3 | Dose reduction | Treatment delay | Hospitalization | Early treatment cessation |
| Cardiotoxicity | 135 (87.1) | 19 (12.3) | 0 (0.0) | 1 (0.6) | - | - | - | 1 (0.6) |
| Constipation | 107 (69.0) | 43 (27.7) | 5 (3.2) | 0 (0.0) | - | - | - | - |
| Diarrhea | 83 (53.5) | 43 (27.7) | 9 (5.8) | 20 (12.9) | 10 (6.5) | 2 (1.3) | 7 (4.5) | 4 (3) |
| Fatigue | 51 (32.9) | 67 (43.2) | 29 (18.7) | 8 (5.2) | - | - | 1 (0.6) | - |
| Hand & foot syndrome | 149 (96.1) | 4 (2.6) | 0 (0.0) | 2 (1.3) | 2 (1.3) | - | - | - |
| Mucositis | 72 (46.5) | 62 (40.0) | 13 (8.4) | 8 (5.2) | 5 (3.2) | 2 (1.3) | 2 (1.3) | - |
| Nausea and/or vomiting | 44 (28.4) | 70 (45.2) | 28 (18.1) | 13 (8.4) | 5 (3.2) | 1 (0.6) | 5 (3.2) | 2 (1.3) |
| Neutropenia | 141 (91.0) | 1 (0.6) | 0 (0.0) | 13 (8.4) | 2 (1.3) | 7 (4.5) | 5 (3.2) | 1 (0.6) |
| Generalized pain | 132 (85.2) | 19 (12.3) | 4 (2.6) | 0 (0.0) | - | - | - | - |
| Peripheral neuropathy | 99 (63.9) | 30 (19.4) | 10 (6.5) | 16 (10.3) | - | - | - | 1 (0.6) |
| Skin toxicity | 124 (80.0) | 23 (14.8) | 7 (4.5) | 1 (0.6) | - | - | - | - |

^a^ Graded according to the National Cancer Institute CTCAE v 4.03 or v 5.0, where grade 0 indicates an absence of the adverse effect and grades 1 to 3 indicating an increase in the frequency and severity of the adverse effect.

Supplementary Table 3: Gene, rs identification number (rs ID), type of variant, base pair change (wild-type (WT), reference > variant (V), alternative), variant (V) allele frequency (%) of the Australian White participant cohort (n = 150-154) and of the wider European population for each single nucleotide polymorphism (SNP) investigated. *P* values from Fisher’s exact tests comparing the variant (V) allele frequency of the participant cohort (n = 150-154) and wider European population.

| Gene | rs ID | Type of variant | Base pair change  WT > V | V allele frequency (%) participant cohort | V allele frequency (%) European^a^ | *P* value ^b^ |
| --- | --- | --- | --- | --- | --- | --- |
| *IL1B* | rs16944 | Upstream | A > G | 63.4 | 65.0 | 0.736 |
|  | rs1143627 | 5’-UTR | C > T | 63.0 | 64.8 | 0.734 |
|  | rs1143634 | Synonymous | G > A | 24.2 | 24.8 | 0.925 |
| *IL2* | rs2069762 | Upstream | A > C | 27.6 | 29.2 | 0.719 |
| *IL6* | rs10499563 | Promoter | T > C | 29.5 | 22.9 | 0.077 |
| *IL10* | rs1800871 | Upstream | A > G | 75.3 | 76.0 | 0.847 |
|  | rs1800896 | Upstream | T > C | 49.3 | 45.3 | 0.370 |
| *IL6R* | rs8192284 / rs2228145 | Missense | A > C | 43.5 | 36.0 | 0.069 |
| *TGFB1* | rs1800469 | Upstream | A > G | 71.2 | 68.8 | 0.594 |
|  | rs11466314 | Upstream | C > T | 0.0^c^ | 0.0 | - |
| *TNF* | rs1800629 | Upstream | G > A | 17.4 | 13.4 | 0.217 |
| *TLR2* | rs3804100 | Synonymous | T > C | 4.2 | 6.4 | 0.364 |
| *TLR4* | rs4986790 | Missense | A > G | 5.3 | 5.7 | > 0.999 |
|  | rs4986791 | Missense | C > T | 5.6 | 5.8 | > 0.999 |
| *MYD88* | rs6853 | 3’-UTR | A > G | 16.7 | 13.1 | 0.251 |
| *MD2 (LY96)* | rs11466004 | Missense | C > T | 1.3 | 2.0 | 0.752 |
| *CASP1 (ICE)* | rs580253 | Synonymous | G > A | 16.6 | 17.6 | 0.829 |
| *CASP5* | rs554344 | Upstream | G > C | 15.0 | 17.6 | 0.441 |
| *OPRM1* | rs1799971 | Missense | A > G | 12.0 | 16.2 | 0.160 |
| *CRP* | rs2794521 | Upstream | C > T | 67.2 | 71.5 | 0.254 |
| *BDNF* | rs6265 | Missense | C > T | 18.9 | 19.7 | 0.918 |
| *DPYD* | rs67376798 | Missense | T > A | 1.0 | 0.7 | > 0.999 |
|  | rs2297595 | Missense | T > C | 11.4 | 11.9 | 0.900 |
|  | rs1801158 | Missense | C > T | 2.3 | 3.1 | 0.801 |
|  | rs3918290 | Splice | C > T | 0.0^c^ | 0.5 | 0.499 |

^a^ from dbSNP 1000 genomes European population, accessed on 31^st^ October 2022; ^b^ FDR-corrected *P* values ranged from 0.92 to 1; ^c^ non-polymorphic SNP.

Supplementary Table 4: Change in logit (95% confidence intervals, *P* values) for each predictor identified in the logistic regression models of adverse events. Positive values indicate increased probability of adverse event and negative values indicate decreased probability of adverse event.

|  | Overall GI toxicity | Diarrhea | Nausea and / or vomiting | Mucositis | Constipation | Neuropathy | Generalized pain | Neutropenia | Hand-foot syndrome | Skin toxicity | Cardiotoxicity | Fatigue |
| --- | --- | --- | --- | --- | --- | --- | --- | --- | --- | --- | --- | --- |
| *IL6*, rs10499563 1 WT |  |  | 1.31  (-0.3 to 2.9, 0.104) |  |  |  |  |  |  |  |  |  |
| *IL6*, rs10499563 2 WT |  |  | 0.32  (-1.2 to 1.8, 0.678) |  |  |  |  |  |  |  |  |  |
| *IL10*, rs1800871 1 WT |  | 0.32  (-0.5 to 1.1, 0.420) |  |  |  |  |  |  | 0.69 (-2 to 3.4, 0.609) |  |  |  |
| *IL10*, rs1800871 2 WT |  | 1.92  (0.1 to 3.7, 0.030) |  |  |  |  |  |  | 39.57  (-15674 to 15753, 0.996) |  |  |  |
| *IL6R*, rs8192284 1 WT |  |  |  |  | 3.27 (0.9 to 5.6, 0.006) |  |  |  |  |  |  |  |
| *IL6R*, rs8192284 2 WT |  |  |  |  | 2.34 (0 to 4.7, 0.048) |  |  |  |  |  |  |  |
| *TGFB1*, rs1800469 1 WT | 1.21  (0.1 to 2.3, 0.028) |  | 1.03  (0.1 to 1.9, 0.021) |  |  |  |  | -0.48 (-2.1 to 1.1, 0.555) |  |  |  |  |
| *TGFB1*, rs1800469 2 WT | 0.11  (-1.4 -to 1.6, 0.876) |  | 1.53  (0.1 to 3.2, 0.061) |  |  |  |  | 3.21 (1.2 to 5.2, 0.001) |  |  |  |  |
| *TLR4*, rs4986790 1 WT |  |  | -17.56  (-2928 to 2893, 0.990) |  |  |  |  | -20.74 (-7933 to 7892, 0.996) | -82.13  (-16207 to 162343, 0.992) |  |  |  |
| *TLR4*, rs4986790 2 WT |  |  | -15.85  (-2927 to 2895, 0.991) |  |  |  |  | -21.11 (-7934 to 7891, 0.996) | -43.59  (-159349 to 159262, 0.999) |  |  |  |
| *MD2* (*LY96*), rs11466004 2 WT |  |  |  | 15.65  (-2384 to 2415, 0.990) |  |  |  |  |  |  |  | 16.43 (-3800 to 3833, 0.993) |
| *CASP5*, rs554344 1 WT |  |  |  |  |  |  |  | -3.78 (-6.8 to -0.7, 0.014) |  |  | -1.11 (-3.4 to 1.2, 0.327) |  |
| *CASP5*, rs554344 2 WT |  |  |  |  |  |  |  | -2.43 (-4.7 to -0.2, 0.030) |  |  | -2.20 (-4.4 to 0, 0.044) |  |
| *BDNF*, rs6265 1 WT |  |  |  |  | -18.65  (-3302 to 3264, 0.991) |  |  | 12.18 (-3513 to 3538, 0.995) |  |  |  |  |
| *BDNF*, rs6265 2 WT |  |  |  |  | -17.16  (-3300 to 3266, 0.992) |  |  | 15.2 (-3510 to 3541, 0.993) |  |  |  |  |
| *DPYD*, rs67376798 2 WT |  |  |  |  |  |  |  |  |  |  |  | 18.2 (-4473 to 4510, 0.993) |
| *DPYD*, rs2297595 1 WT |  |  |  |  | 35.58  (-4607 to 4679, 0.988) |  |  |  |  |  |  |  |
| *DPYD*, rs2297595 2 WT |  |  |  |  | 35.09  (-4608 to 4678, 0.988) |  |  |  |  |  |  |  |
| Treatment hospital, Royal Adelaide Hospital | -0.92  (-1.8 to 0, 0.046) | -0.8  (-1.6 to 0, 0.055) |  | -1.65  (-2.5 to  -0.9, <0.0001) |  | -1.76 (-3.2 to -0.4, 0.012) |  |  |  |  | -18.47 (-2862 to 2826, 0.990) | -1.7 (-2.7 to -0.7, 0.0004) |
| Colorectal cancer |  | 1.57  (0.8 to 2.4, 0.0001) |  |  |  | 4.3 (2.8 to 5.8, <0.0001) |  |  |  |  |  |  |
| Gastric cancer |  | 0.92  (-0.6 to 2.5, 0.237) |  |  |  | 2.12 (0.3 to 3.9, 0.017) |  |  |  |  |  |  |
| 5-FU+ chemotherapy |  |  |  |  | 1.66 (0.1 to 3.2, 0.028) | 4.44 (2.4 to 6.4, <0.0001) | 16.99  (-2660 to 2694, 0.990) |  | -38.37  (-16103 to 16026, 0.996) |  |  |  |
| Capecitabine chemotherapy |  |  |  |  | -0.19 (-2.9 to 2.5, 0.891) | 2.24 (0.4 to 4.1, 0.016) | 16.86  (-2660 to 2694, 0.990) |  | 19.43  (-10476 to 10515, 0.997) |  |  |  |
| Age at treatment |  |  |  |  |  |  |  |  |  |  | 0.04 (0 to 0.1, 0.161) |  |
| Body surface area |  |  | -1.58  (-3.6 to 0.5, 0.121) |  |  |  |  |  |  |  |  |  |
| Alcohol use |  |  |  |  | 2.51 (0.7 to 4.3, 0.006) |  | 1.77 (-0.4 to 3.9, 0.097) |  |  |  |  |  |
| Arthritis |  |  |  |  |  |  |  |  |  |  |  | -2.66 (-5.3 to 0, 0.042) |
| Hypothyroidism |  |  |  |  |  |  | 2.17 (-0.4 to 4.7, 0.092) |  |  |  |  |  |
| Type 2 diabetes mellitus |  |  |  |  |  |  |  |  |  |  | 2.78 (1.1 to 4.4, 0.0008) |  |
| Non-smoker |  |  |  |  | 0.13 (-1.1 to 1.3, 0.834) |  |  |  |  | -2.01  (-3.6 to -0.4, 0.010) |  | -0.96 (-1.9 to 0, 0.046) |
| Smoker |  |  |  |  | 1.58 (0.1 to 3.1, 0.038) |  |  |  |  | -0.99  (-2.6 to 0.6, 0.218) |  | 0.81 (-1 to 2.6, 0.366) |

Abbreviations: 5-fluorouracil (5-FU)+ chemotherapy - 5-FU in combination therapies of ECF, EOF, FEC and FOLFOX; capecitabine chemotherapy – alone or with oxaliplatin; 1 WT – 1 copy wild-type (WT, reference) allele; 2 WT – 2 copies wild-type (WT, reference) alleles.
